# On-Wafer Gate Screening Test for Improved Pre-Reliability in p-GaN HEMTs

**DOI:** 10.3390/mi16080873

**Published:** 2025-07-29

**Authors:** Giovanni Giorgino, Cristina Miccoli, Marcello Cioni, Santo Reina, Tariq Wakrim, Virgil Guillon, Nossikpendou Yves Sama, Pauline Gaillard, Mohammed Zeghouane, Hyon-Ju Chauveau, Maria Eloisa Castagna, Aurore Constant, Ferdinando Iucolano, Alessandro Chini

**Affiliations:** 1 Department of Engineering “Enzo Ferrari”, Università di Modena e Reggio Emilia, 41125 Modena, Italy; alessandro.chini@unimore.it; 2 STMicroelectronics, 95121 Catania, Italy; cristina.miccoli@st.com (C.M.); marcello.cioni@st.com (M.C.); santo.reina@st.com (S.R.); mariaeloisa.castagna@st.com (M.E.C.); ferdinando.iucolano@st.com (F.I.); 3 STMicroelectronics, 37071 Tours, France; tariq.wakrim@st.com (T.W.); virgil.guillon@st.com (V.G.); nossikpendouyves.sama@st.com (N.Y.S.); pauline.gaillard@st.com (P.G.); mohammed.zeghouane@st.com (M.Z.); hyon-ju.chauveau@st.com (H.-J.C.); aurore.constant@st.com (A.C.)

**Keywords:** HEMTs, p-GaN, gate reliability, TCAD, surface treatment

## Abstract

In this paper, preliminary gate reliability of p-GaN HEMTs under high positive gate bias is studied. Gate robustness is of great interest both from an academic and industrial point of view; in fact, different tests and models can be explored to estimate the device lifetime, which must meet some minimum product requirements, as specified by international standards (AEC Q101, JESD47, etc.). However, reliability characterizations are usually time-consuming and are performed in parallel on multiple packaged devices. Therefore, it would be useful to have a faster method to screen out weaker gate trials, already on-wafer, before reaching the packaging step. For this purpose, a room-temperature stress procedure is presented and described in detail. Then, this screening test is applied to devices with a reference gate process, and, as a result, high gate leakage degradation is observed. Afterwards, a different process implementing a dielectric layer between p-GaN and gate metal is evaluated, highlighting the improved behavior during the stress test. However, it is also observed that devices with this process suffer from very high drain leakage, and this effect is then studied and understood through TCAD (technology computer-aided design) simulations. Finally, the effect of a surface treatment performed on the p-GaN is analyzed, showing improved gate pre-reliability while maintaining low drain leakage.

## 1. Introduction

GaN-based HEMTs are emerging wide-bandgap semiconductor devices well-suited to RF (e.g., 5G power amplifier modules), power (e.g., automotive, data center, and consumer power supply), and space (e.g., sensors and detectors in harsh environments) applications [1,2]. These devices exhibit superior features, including high breakdown voltage, low capacitance, high frequency, and high power density [3,4,5,6]. In the case of power applications, where normally-off operation is usually preferred, several solutions have been proposed in the literature for obtaining the 2DEG depletion at zero gate bias [7]. One of the most promising and already commercially available solutions is the p-GaN HEMT, where a p-doped GaN layer is grown on top of the AlGaN/GaN stack and patterned to define the gate region [8,9]; then, a gate metal is deposited on the p-GaN to form either an ohmic or a Schottky contact [10].

In order to obtain the optimal on-resistance and saturation current with limited gate leakage, the gate terminal of Schottky p-GaN HEMTs is typically driven at around +6 V with respect to the source terminal. However, devices are required to sustain this voltage level during their operating lifetime. Therefore, a key aspect in the device development and qualification flow consists of reliability stress tests that are useful to estimate the degradation of the performance under certain conditions of voltage/temperature/pressure/humidity, etc. [11] and to ensure that the large majority of fabricated devices are not going to fail during their functioning. Given the fact that power HEMTs are switching between an ON state and an OFF state, both conditions can induce some kind of failure or degradation, and must be studied separately for a better understanding of the phenomena involved. In particular, when the transistor is switched ON, the forward gate bias applied to the device (i.e., +6 V) may induce gate leakage degradation and eventually failure. Actually, there are also some other bias conditions (e.g., high drain-source voltages) that can cause gate reliability issues; however, they will not be discussed further in this work. Under moderately high positive gate bias, it is demonstrated by TCAD (technology computer-aided design) simulations that the main stressed region, in terms of high electric field, is the gate metal corner interface with the p-GaN [12,13], but there can also be other sources of degradation, involving electron injection from the AlGaN barrier [14,15]. However, the time-to-failure dependence on the applied gate bias is not easy to determine since the acceleration factor can be modified by the specific physical gate leakage conduction mechanism involved in each different bias range (e.g., low VGS, medium VGS, high VGS) that can lead to complex modeling through modified E-models or power laws [16,17], requiring many trials and a lot of time and effort with the final goal of achieving at least, e.g., 10 years of expected lifetime (which is a usual customer requirement). Following the indications of the international standards (AEC Q101 [18], JESD47 [19], JEP180 [20], etc.), it can also be noticed that the most common gate stress test is represented by the HTGB (high-temperature gate bias). This test is typically performed at VGS = 6 V and a temperature of 150 °C for 1000 h in the case of p-GaN HEMTs with nominal driving at VGS = 6 V. Since all of these stress tests are quite time-consuming, they are usually performed in parallel on many packaged devices through dedicated equipment. Therefore, there may be a need for an alternative method for an easier and faster on-wafer early screening of p-GaN gate processes through some accelerated gate stress procedure, able to give a first idea of the gate robustness.

The content of this paper focuses on the following key aspects. Descriptions of the device structure and experimental stress test setup are reported in Section 2. In Section 3, DC characterizations and the outputs of the proposed gate stress test are presented for a reference p-GaN HEMT process. These results are then compared to the ones obtained for two gate process variations, showing how it is possible to reach a significant improvement in the gate robustness by applying an extra surface treatment step on the p-GaN. Finally, a summary of the main results discussed in the paper is provided in Section 4.

## 2. Device Structure and Stress Test

**Figure 1:**
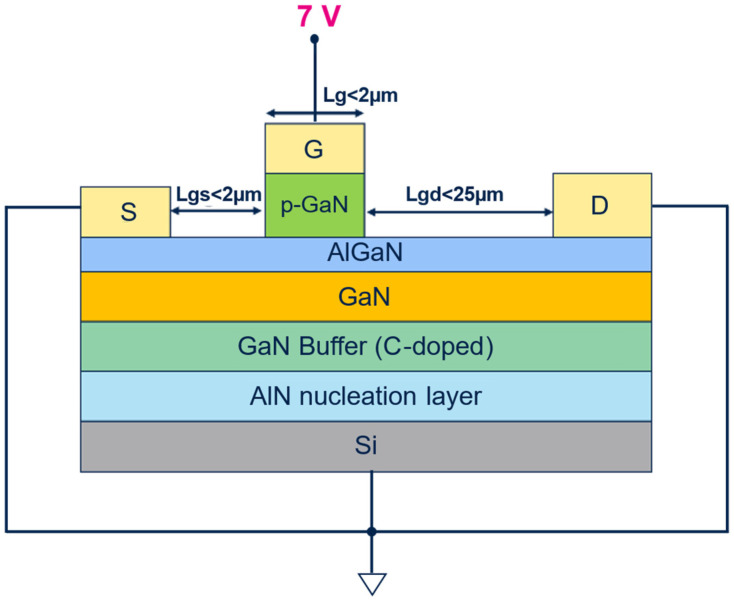
Schematic view of the cross-section of the device including the bias conditions of the proposed gate stress conditions.

Devices Under Test (DUTs) are 650 V-rated p-GaN HEMTs for power applications, with Mg-doping in p-GaN approximately 1 × 10^19^ cm^−3^ and p-GaN thickness in the range of 60–120 nm, grown on a p-type low-resistive Si substrate using a metal organic–chemical vapor deposition (MOCVD) system. The schematic cross-section of a typical DUT is depicted in Figure 1, with an indication of the main lateral dimensions. Devices analyzed in the following are characterized by a large active area (a few mm^2^), which makes it easier to spot possible failures within a reduced stress time window [21,22].

The bias conditions for the proposed stress test are reported in Figure 1 as well, where it is shown that all the terminals of the device are grounded except for the gate one, which is biased at a constant voltage, similarly to what happens in the case of HTGB. However, in this case, the idea is to apply +7 V on the gate terminal for a total stress time of 10,000 s at room temperature (25 °C). This 10,000 s stress duration has been chosen as an acceptable trade-off between a minimum reasonable observability time for potential stress-induced gate failures and the repeatability of the stress procedure for a considerable number of devices. In fact, in order to acquire meaningful statistical data, 24 devices per wafer are stressed in the above-described conditions.

The bias conditions are experimentally applied on several devices directly on the wafer, thanks to a B1505A parameter analyzer [23] and a probe system [24].

During the proposed room-temperature gate bias (“RTGB”) stress test, the gate leakage is probed every 10 s in order to monitor the device behavior during the stress time. Moreover, transfer characteristics and leakage measurements before and after gate stress are performed to evaluate possible drifts on the main relevant parameters (i.e., on-resistance, threshold voltage, gate and drain leakage). The test sequence applied to each DUT is listed below (the entire sequence is performed at a temperature of 25 °C):

1. Pre-stress transfer characteristic;
2. Pre-stress gate leakage;
3. Pre-stress drain leakage;
4. RTGB (7 V, duration = 10^4^ s);
5. Post-stress transfer characteristic;
6. Post-stress gate leakage;
7. Post-stress drain leakage.

It is important to underline that the chosen stress conditions are meant to accelerate the degradation on the p-GaN gate of the devices, and in particular (i) +7 V is a more challenging condition than the nominal operating voltage (+6 V) but at the same time it is not too far, hence a similar gate conduction mechanism can be assumed, and (ii) 25 °C is usually a more stressful temperature than 150 °C, given the negative temperature acceleration factor (AF) of the p-GaN under positive gate bias stress [25], which is generally linked to the fact that, for example, avalanche breakdown phenomena (such as impact ionization) are penalized at higher temperature. While there can be other stress phenomena becoming increasingly significant at higher temperatures (like ion migration/diffusion, Frenkel defect formation, defect generation, or material degradation, etc.), the negative temperature AF was confirmed for the DUTs through stress measurements at different temperatures. Therefore, the chosen stress conditions are accelerating the degradation while also staying fairly close to the actual operating bias conditions, thus reducing the risk of additional physical degradation mechanisms occurring, e.g., at higher gate voltages and induced by strong hole injection [26,27], which can modify the lifetime and lead to unreliable results.

## 3. Results and Discussion

In this section, the proposed accelerated gate stress test is applied to three different families of DUTs, characterized by different processing of the p-GaN region. Starting with a reference process, two improved solutions are then analyzed in terms of gate pre-reliability and DC performance.

### 3.1. Reference Process

**Figure 2:**
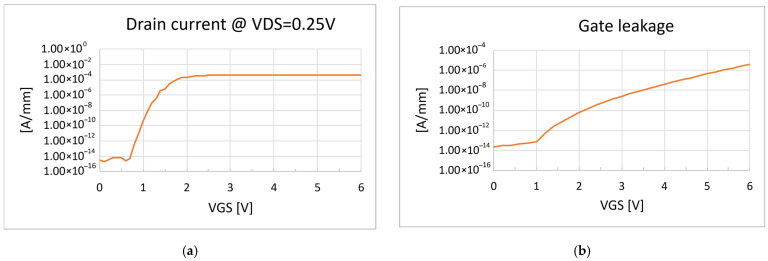
(**a**) Typical linear transfer characteristic of DUTs fabricated with reference process; (**b**) typical gate leakage of DUTs fabricated with reference process.

**Figure 3:**
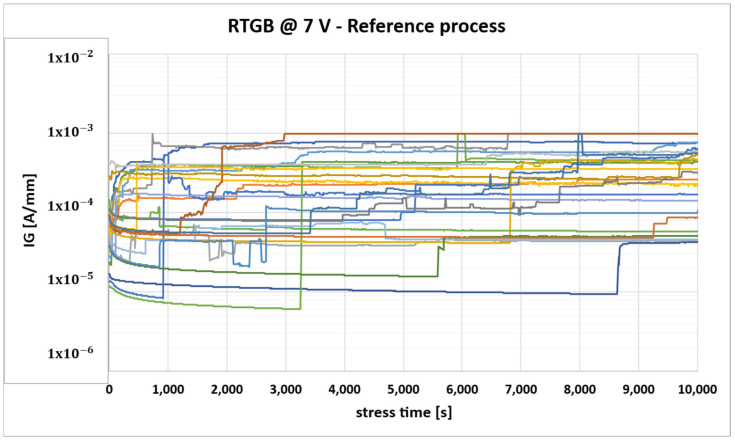
Gate leakage monitoring during RTGB stress for DUTs fabricated with reference process (each color identifies the behavior of one of the stressed DUTs).

DUTs fabricated with a reference process have been characterized in DC (Figure 2) and then stressed with the RTGB stress to test their endurance. A relevant gate leakage degradation was observed during the monitoring (Figure 3), with some devices showing a worsening with increasing stress time, consistently with, e.g., a percolation-driven phenomenon [28,29].

The observed behavior during RTGB already suggests poor gate stability in the case of a moderately large positive voltage; thus, there is no need to perform additional gate reliability tests, like HTGB, on packaged samples, because it is certain that devices would not pass those stress tests.

### 3.2. Interdielectric Process

**Figure 4:**
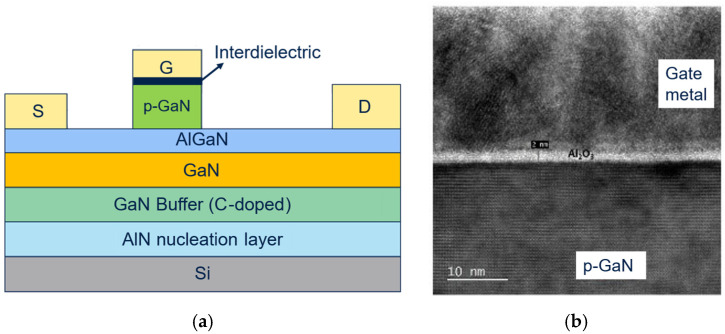
(**a**) Schematic view of the cross-section of the device with interlayer dielectric between p-GaN and gate metal; (**b**) transmission electron microscopy (TEM) photograph of the gate module.

In order to mitigate the gate leakage degradation, a variation in the process flow, consisting of the ex situ atomic layer deposition (ALD) of a dielectric interlayer (namely Al_2_O_3_) of about 0.5–5 nm between p-GaN and gate metal, was implemented, as shown in Figure 4. Indeed, the introduction of this additional interdielectric layer can improve the insulation of the gate and have a passivation effect on the p-GaN surface, obtaining an MIS (metal–insulator–semiconductor) gate structure.

**Figure 5:**
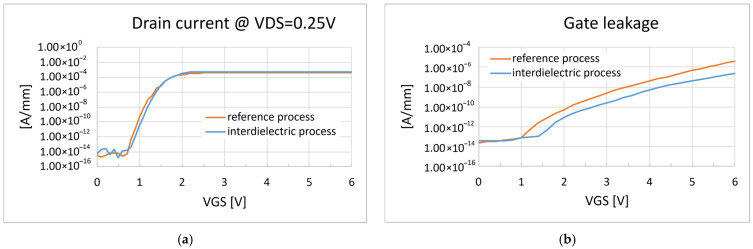
(**a**) Comparison of the transfer characteristic curves; (**b**) comparison of the gate leakage curves.

DC characterizations of devices with such modification are shown in Figure 5, where the reference devices are compared to devices processed with the interdielectric approach.

**Figure 6:**
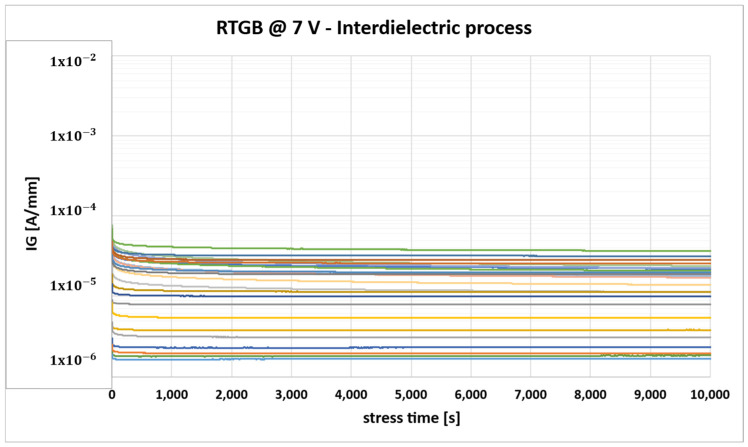
Gate leakage monitoring during RTGB stress for devices with interlayer dielectric (each color identifies the behavior of one of the stressed DUTs).

The comparison of the DC characteristics reported above shows no significant variations between the two different gate implementations, apart from a small reduction of the gate leakage in the case of the interdielectric. More importantly, the introduction of the interdielectric layer proved to be effective in improving the gate reliability of all the tested devices when performing the RTGB stress, as shown in Figure 6.

**Figure 7:**
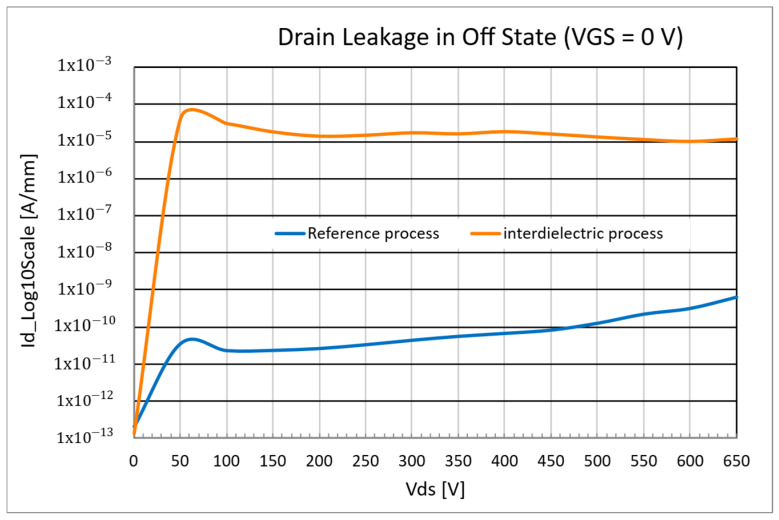
Drain leakage comparison between reference process and inter-dieletric process.

Nevertheless, even if the threshold voltage in the linear region is not affected by the process, it was observed that the introduction of the interdielectric layer leads to an increase in the drain leakage current, as shown in Figure 7, due to a worse control of the electrostatic potential under the p-GaN region (“channel potential”). This phenomenon was verified through TCAD simulations, detailed in the following.

**Figure 8:**
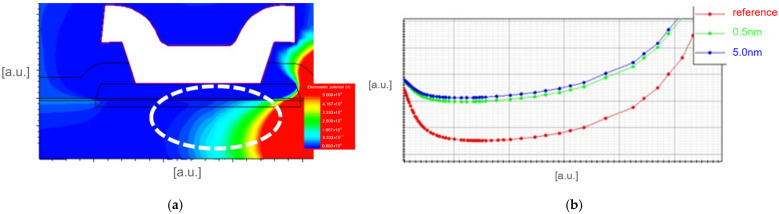
TCAD simulation results: (**a**) 2D distribution of the electrostatic potential close to the gate module (VGS = 0 V, VDS = 650 V); (**b**) Horizontal cut of the electrostatic potential under the p-GaN region (white dotted circled area) in the case of reference process, interdielectric process with 0.5 nm, interdielectric process with 5 nm.

TCAD simulations using SYNOPSYS tools were carried out to investigate the physical mechanisms near the p-GaN gate region. The p-GaN HEMT structure was reproduced through Sentaurus Process, while the device simulation was performed thanks to Sentaurus Device [30], including physical models for GaN-based devices. DUT was simulated in OFF state (V_GS_ = 0 V, V_DS_ = 650 V), and as reported in Figure 8, the potential under the p-GaN region, at the drain-side, is higher in the case of an interdielectric between p-GaN and gate metal, which results in a lower energy barrier (between source and drain) for the electrons.

**Figure 9:**
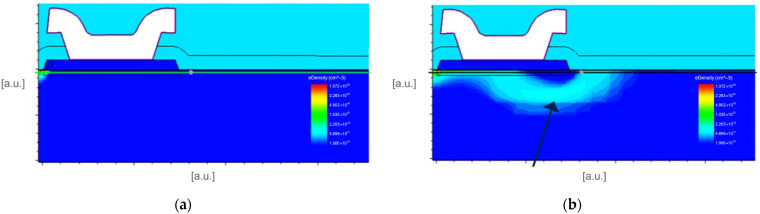
TCAD simulation results at VGS = 0 V and VDS = 650 V: (**a**) 2D distribution of the electron density for the reference process; (**b**) 2D distribution of the electron density for the interdielectric process (the arrow highlights the region with higher density).

This phenomenon can be explained by the fact that the dielectric layer introduces an additional insulating barrier between the gate metal and p-GaN, which produces a redistribution of the electric field, given the different boundary conditions, mainly localized at the drain side of the gate. However, at low drain-source bias, the electric field is limited and, therefore, the dielectric layer does not significantly perturb the electrostatic potential. Instead, at higher bias, the electric field at the drain edge of the p-GaN/AlGaN interface becomes high, and the local distortion of the potential distribution can facilitate a leakage path. Therefore, this variation in the electrostatic potential is the root cause of the formation of an electron current path between the source and drain, even with zero gate bias (Figure 9).

### 3.3. Surface Treatment

**Figure 10:**
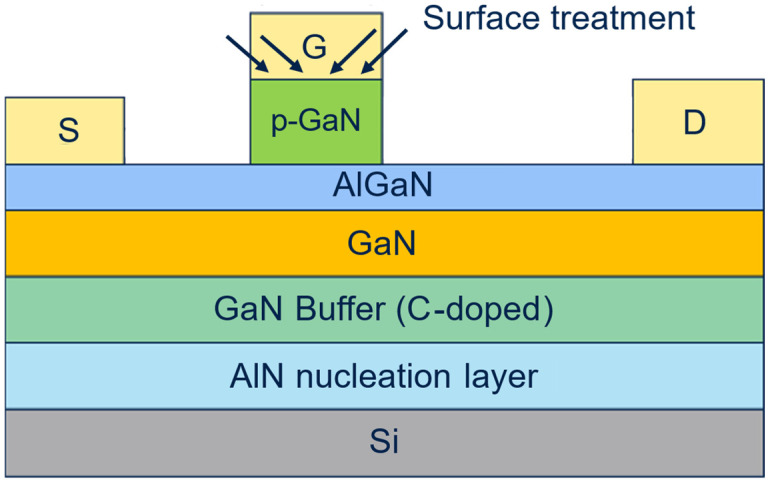
Sketch of the DUTs fabricated with the addition of the surface treatment step.

The third option was tried to improve the gate robustness without the limitations observed in the interdielectric process. In this case, starting from the reference process, a different solution is proposed. In particular, an extra step of p-GaN surface treatment is performed during the process flow (Figure 10). In particular, this process consists of a dry treatment performed at room temperature where the p-GaN surface is exposed to ozone (O_3_), in order to obtain a cleaner and passivated surface and improve the interface quality, which is critical for enhancing long-term reliability.

**Figure 11:**
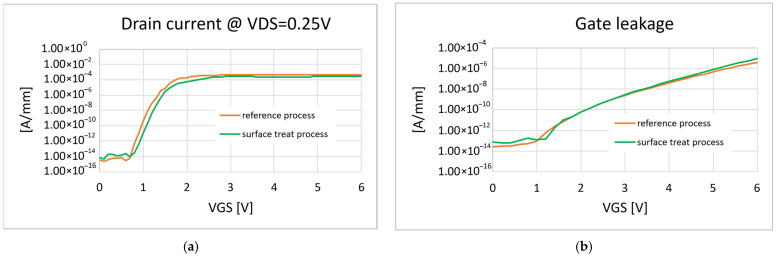
(**a**) Comparison of the transfer characteristic curves; (**b**) comparison of the gate leakage curves.

As in the previous cases, a small DC characterization was performed on the devices, and the results (compared to the reference process) are reported in Figure 11.

**Figure 12:**
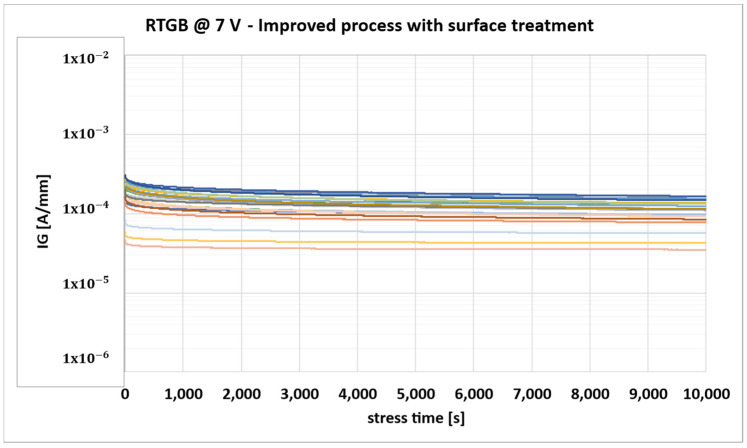
Gate leakage monitoring during RTGB stress for devices with surface treatment (each color identifies the behavior of one of the stressed DUTs).

Then, the RTGB stress test was performed on the DUTs fabricated with said process, and the results are shown in Figure 12.

**Figure 13:**
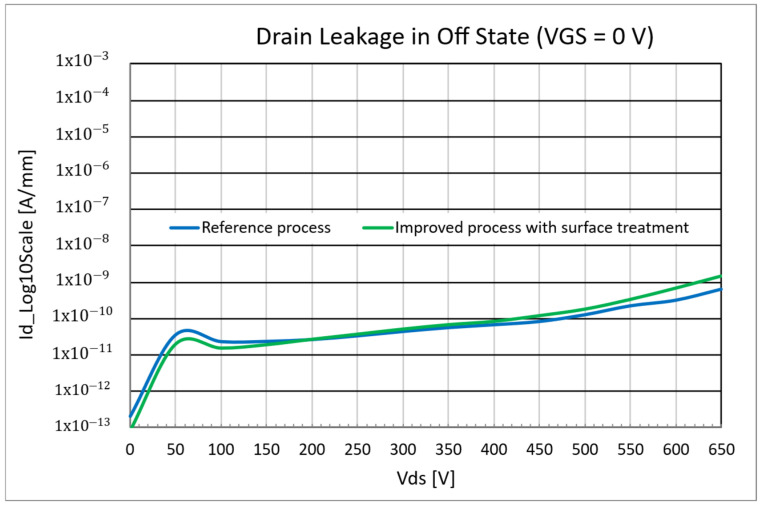
Drain leakage comparison between reference process and improved process with surface treatment.

As in the previous case, all the tested devices showed no degradation of the gate leakage during the stress. However, for this improved process, it was observed that low drain leakage was maintained up to high VDS, with no increase or worsening with respect to the reference process (Figure 13).

**Figure 14:**
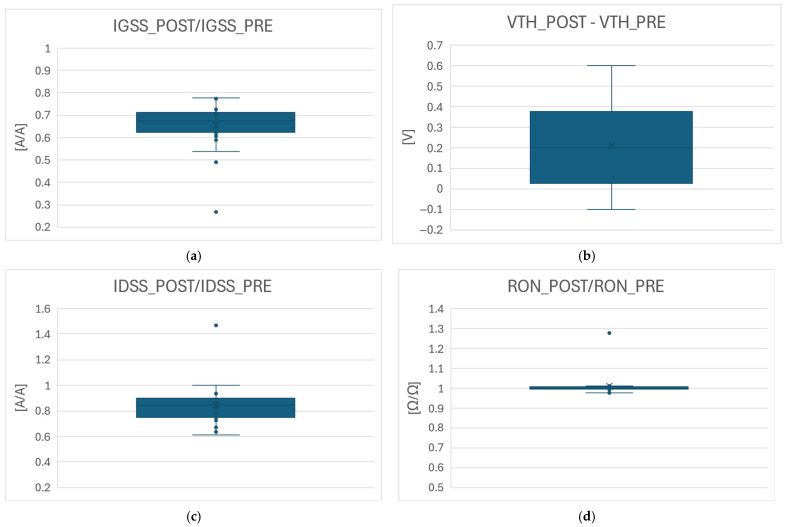
Parametric drift analysis after gate stress test on DUTs with surface treatment: (**a**) ratio of gate leakage after stress over before stress value; (**b**) shift of threshold voltage after stress with respect to before stress value; (**c**) ratio of drain leakage after stress over before stress value; (**d**) ratio of on-resistance after stress over before stress value.

In this case, given the fact that all the devices passed the RTGB test, a drift analysis on the main DC parameters was performed (Figure 14). As can be seen, after the gate stress, no significant degradation was observed on the ON state (on-resistance, gate leakage) or OFF state (drain leakage) parameters, while the threshold voltage, V_TH_ (semi-ON state parameter), is slightly affected (small increase).

Therefore, it can be concluded that the surface treatment of the p-GaN can be of paramount importance for the gate’s early reliability and must be carefully engineered to obtain the best results. Instead, the introduction of a dielectric layer between p-GaN and gate metal is a risky option, since it degrades the channel control at zero gate bias in the case of high drain-source voltage. However, since both solutions were able to improve preliminary gate reliability under positive gate bias stress conditions, it can be assumed that, in the reference process, the failure region is located mainly at the metal/p-GaN Schottky interface and it can be probably related to impurities or other contaminants on the p-GaN surface (dedicated failure analyses are needed to further investigate the root cause but they are out of the scope of this work). In conclusion, the proposed RTGB test can be a quick and useful tool for benchmarking on-wafer devices fabricated with a specific process flow.

## 4. Conclusions

Gate preliminary reliability of p-GaN HEMTs under positive gate bias has been studied through a room-temperature on-wafer accelerated stress test (RTGB), proposed to speed up the comparison of different gate module trials. Different gate processes have been presented and compared, highlighting the drawbacks and advantages of each approach. First, DUTs fabricated with a reference process have been tested, and the experimental characterizations have shown poor gate reliability behavior. Then, a first process variation including a dielectric layer between p-GaN and gate metal has been proposed for improved gate robustness. Devices with this process have been stressed, and the results have shown improved gate reliability performance, at the cost of a significantly increased drain leakage. This effect has also been examined through the help of TCAD simulations, which have shown how the potential distribution under the p-GaN region is negatively impacted by the introduction of a dielectric layer sandwiched between p-GaN and gate metal. Consequently, the drain leakage increases in this condition. An additional solution, consisting of an extra surface treatment step on the p-GaN, has been finally shown and described, underlying its good reliability performance under the considered stress conditions. This approach also allows good control of the channel region under the p-GaN, in terms of low drain leakage, up to elevated drain-source bias.

## Data Availability

The data presented in this study are available on request from the corresponding author.
